# Possible silent hypoxemia in a COVID-19 patient: A case report

**DOI:** 10.1016/j.amsu.2020.11.053

**Published:** 2020-11-24

**Authors:** Siswanto, Munawar Gani, Aditya Rifqi Fauzi, Ririn Enggy Yuliyanti, Maria Patricia Inggriani, Bagus Nugroho, Denny Agustiningsih, Gunadi

**Affiliations:** a Department of Physiology, Faculty of Medicine, Public Health and Nursing, Universitas Gadjah Mada/UGM Academic Hospital, Yogyakarta, 55291, Indonesia; b Pulmonology Division, Department of Internal Medicine, Faculty of Medicine, Public Health and Nursing, Universitas Gadjah Mada/Dr. Sardjito Hospital, Yogyakarta, 55281, Indonesia; c Pediatric Surgery Division, Department of Surgery, Faculty of Medicine, Public Health and Nursing, Universitas Gadjah Mada/Dr. Sardjito Hospital, Yogyakarta, 55281, Indonesia; d Panti Rapih Hospital, Yogyakarta, 55223, Indonesia; e Department of Physiology, Faculty of Medicine, Public Health and Nursing, Universitas Gadjah Mada, Yogyakarta, 55281, Indonesia

**Keywords:** ARDS, COVID-19, Early sign of deterioration, Respiratory failure with severe hypoxia, Silent hypoxemia

## Abstract

**Introduction:**

It has been hypothesized that silent hypoxemia is the cause of rapid progressive respiratory failure with severe hypoxia that occurs in some COVID-19 patients without warning.

**Presentation of case:**

A 60-year-old male presented cough without any breathing difficulty. Vital signs showed blood pressure 130/75 mmHg, pulse 84x/minute, respiratory rate (RR) 21x/minute, body temperature 36.5C, and oxygen saturation (SpO2) 75% on room air. RT-PCR for COVID-19 were positive. On third day, he complained of worsening of breath shortness, but his RR was still normal (22x/minute) with SpO2 of 98% on 3 L/minute oxygen via nasal cannula. On fifth day, he experienced severe shortness of breath with RR 38x/minute. He was then intubated using a synchronized intermittent mandatory ventilation. Blood gas analysis showed pH 7.54, PaO2 58.9 mmHg, PaCO2 31.1 mmHg, HCO3 26.9mEq/L, SaO2 94.7%, FiO2 30%, and P/F ratio 196 mmHg. On eighth day, his condition deteriorated with blood pressure 80/40 mmHg with norepinephrine support, pulse 109x/minute, and SpO2 72% with ventilator. He experienced cardiac arrest and underwent basic life support, then resumed strained breathing with return of spontaneous circulation. Blood gas analysis showed pH 7.07, PaO2 58.1 mmHg, PaCO2 108.9 mmHg, HCO3 32.1mEq/L, SaO2 78.7%, FiO2 90%, and P/F ratio 65 mmHg. Three hours later, he suffered cardiac arrest again and eventually died.

**Discussion:**

Possible mechanisms of silent hypoxemia are V/Q mismatch, intrapulmonary shunting, and intravascular microthrombi.

**Conclusions:**

Silent hypoxemia might be considered as an early sign of deterioration of COVID-19 patients, thus, physician may be able to intervene early and decrease its morbidity and mortality.

## Highlights

• Some patients with COVID-19 might experience rapid deterioration without warning.
• Silent hypoxemia might be considered as an early clinical sign of deterioration of patients with COVID-19.
• Several hypotheses of silent hypoxemia have been proposed, including V/Q mismatch, intrapulmonary shunting and endothelial injury.

## 1 Introduction

The SARS-CoV-2 virus that causes Coronavirus Disease 2019 (COVID-19) was declared a pandemic since the World Health Organization (WHO) decree on March 11, 2020, and has infected more than 54 million people and caused more than 1.3 million deaths as of November 14, 2020 [1,2].

The early sign of severe disease of SARS-CoV-2 infection is pneumonia with respiratory failure, similar to Acute Respiratory Distress Syndrome (ARDS). Although hypoxic acute respiratory failure causes an increase in respiratory rate (RR), in some patients, a persistent normal RR was found and inconsistent with the severity of hypoxia. Some patients with COVID-19 reported experiencing rapid deterioration without warning. This reaction might be caused by ‘silent hypoxemia’. Research has shown that failure of pulmonary oxygen diffusion causes a gradual decrease in oxygen saturation [3,4]. Here, we reported one COVID-19 case with the possibility of silent hypoxemia.

## 2 Presentation of case

**Fig. 1:**
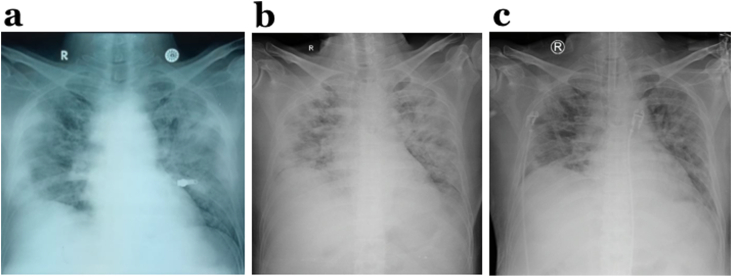
Chest x-rays: a) on the admission day indicated bilateral pneumonia, which is not compatible with the relatively slight clinical manifestations of patient, b) on the third day, and c) on the eight day also showed bilateral pneumonia.

**Fig. 2:**
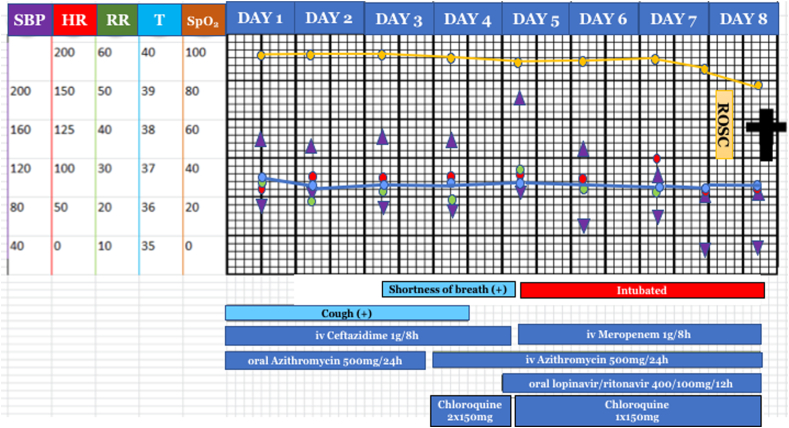
Clinical picture and disease progression of case. ROSC, return of spontaneous circulation; iv, intravenous; †, died. Y-axis scale: SBP, systolic blood pressure (mmHg, violet triangle); HR, heart rate (times/min, red circle); RR, respiratory rate (times/min, green circle); T, temperature (°C, blue circle); SpO_2_, oxygen saturation (%, yellow circle). (For interpretation of the references to colour in this figure legend, the reader is referred to the Web version of this article.)

A 60-year-old Javanese male came to outpatient clinic in our hospital with complaints of cough that was felt for two weeks before admission without any breathing difficulty. Complaints were accompanied by fever, runny nose and sore throat. He had a comorbid condition of uncontrolled diabetes mellitus (DM). He was not a smoker and had no history of chronic pulmonary disease. His vital signs examination showed blood pressure 130/75 mmHg, pulse 84 times per minute, normal respiratory rate (RR) of 21 times per minute, body temperature 36.5C, 75% oxygen saturation on room air. His body mass index is 28.04, categorized as overweight. On physical examination, an increase in vesicular sounds and crackles in both lungs were identified. Laboratory tests showed an increase in C-reactive protein (CRP), neutrophil-lymphocyte ratio, aspartate transaminase (AST), and alanine aminotransferase (ALT) of 140 mg/L, 8.7, 88 μ/L, and 116 μ/L, respectively. Chest x-ray showed bilateral pneumonia (Fig. 1). Sputum and GeneXpert tests were performed, and the results were negative for tuberculosis infection. The nasopharyngeal and oropharyngeal swab real-time polymerase chain reaction tests for COVID-19 were positive. After admission, the patient received antibiotics and antiviral therapy based on the COVID-19 Prevention and Control guidelines by the Indonesian Ministry of Health [5], namely intravenous azithromycin 500mg once daily, oral lopinavir/ritonavir 400/100mg twice daily, oral chloroquine sulfate 150 mg twice a day, intravenous meropenem 1 gr thrice daily, and medications for his DM (Fig. 2). On the third day of treatment, the patient complained of worsening of shortness of breath, but his RR was still normal with 22 times per minute with SpO2 of 98% on 3 L/minute oxygen via nasal cannula. On the fifth day of treatment, the patient experienced severe shortness of breath with a RR of 38 times per minute. The patient was then intubated using a mechanical ventilator with synchronized intermittent mandatory ventilation. The patient was not in a prone position. Moreover, continuous positive airway pressure was not utilized in this patient as a bridging intervention to mechanical ventilation. His blood gas analysis showed respiratory alkalosis (pH 7.54, PaO2 58.9 mmHg, PaCO2 31.1 mmHg, HCO3 26.9 mEq/L, SaO2 94.7%, FiO2 30%, P/F ratio 196 mmHg, indicating moderate ARDS). On the following day, his blood gas analysis showed compensated respiratory alkalosis (pH 7.45, PaO2 64.6 mmHg, PaCO2 42.9, HCO3 29.9 mEq/L, SaO2 93.6%, FiO2 60%, P/F ratio of 111.5 mmHg, indicating moderate ARDS). On the eighth day of treatment, his condition deteriorated starting in the morning, with blood pressure 80/40 mmHg with norepinephrine support, pulse 109 times per minute, and 72% SpO2 with ventilator. In the afternoon, the patient experienced cardiac arrest and underwent basic life support, then resumed strained breathing with return of spontaneous circulation. His laboratory results showed leucocyte count of 21.33x103/μL, AST of 112 μ/L, ALT of 96 μ/L, blood urea nitrogen of 41.5 mg/dL, creatinine of 3.36 mg/dL, and CRP of 140 mg/L. Blood gas analysis showed severe respiratory acidosis (pH 7.07, PaO2 58.1 mmHg, PaCO2 108.9 mmHg, HCO3 32.1 mEq/L, SaO2 78.7%, FiO2 90%, P/F ratio of 65 mmHg, indicating severe ARDS) (Fig. 2). Three hours later, he suffered cardiac arrest again, but was unable to be resuscitated. The patient eventually died.

## 3 Discussion

Here, we discuss a case with COVID-19 with the possibility of silent hypoxemia. Hypoxemia itself is defined as a potential life-threatening condition characterized by a decrease in arterial PO2 below the normal value. Hypoxemia documentation can be done by checking pulse oximetry and arterial blood gas analysis. Hypoxemia occurs when PaO2 is less than 80 mmHg, and severe hypoxemia is when it is less than 60 mmHg. There are four main factors that can impair pulmonary gas exchange and cause hypoxemia when breathing room water is at sea level: hypoventilation, diffusion limitation, shunt, and ventilation-perfusion (V/Q) mismatch [[7], [8], [9]].

This unusual ‘silent hypoxemia’ phenomenon showed it is possible that the virus has an idiosyncratic effect on the respiratory control system. Angiotensin-converting-enzyme 2 (ACE2) receptors are highly expressed in the carotid bodies, which are also the same sites where chemoreceptors sense oxygen. ACE2 receptors are also widely expressed in the nasal mucosa. Symptoms of anosmia-hyposmia are experienced by a third of patients with COVID-19, and the olfactory bulb can be the entrance of the virus to the brain and may also play a role in depressed dyspnea response [10,11].

Moreover, ACE2 counteracts the physiological functions of ACE and results in the activation of the renin-angiotensin-aldosterone system (RAAS) related to blood pressure regulation through the conversion of Angiotensin I to Angiotensin II and the electrolyte homeostasis. During the hypoxia condition, Angiotensin II induces vasoconstriction to improve the V/Q mismatch, however, at the same time, it also stimulates the pro-fibrotic effect, and both effects are aggravated by the concomitant upregulation of ACE2 [[10], [11], [12]].

Gattinoni et al. [13] suggested two primary phenotypes of hypoxemia in patients with COVID-19: type L and type H. Type L is caused by loss of respiratory regulation and loss of hypoxic vasoconstriction. The condition of hypoxemia is due to an increase in minute ventilation, mainly by increasing the tidal volume (up to 15–20 ml/kg). Meanwhile, type H is a transition from type L, which is associated with a more negative intrathoracic inspiratory pressure. Diffuse pulmonary microvascular thrombosis is also believed to be the cause of hypoxemia in patients with COVID-19 [14].

Pneumonia analysis of COVID-19 shows that the air sacs in the lungs of patients are not filled with fluid or pus as in pneumonia infections in general but instead the virus causes the water sacs to collapse, thereby reducing the oxygen level and causing hypoxia in the patient, but the reaction still enhances the normal lung ability to expel carbon dioxide. Since carbon dioxide removal is still effective, patients do not feel shortness of breath [15]. Another mechanism proposed is intrapulmonary shunting. Infection causes interstitial edema, loss of surfactant and superimposed pressure, which induces alveolar collapse and substantial fraction of cardiac output perfusing non-aerated lung tissue. Over time, increased edema will increase lung weight, alveolar collapse, and dependent atelectasis, leading to increased shunting [16].

Endothelial injury as a central hallmark in the pathogenesis of COVID-19 is thought to also play a role in the mechanism of silent hypoxemia. SARS-CoV-2 can directly infect lung capillary endothelial cells expressing ACE2. Endothelial injury and acute inflammation provoke the formation of intravascular microthrombi. Lung autopsies in patients after severe disease have shown diffuse alveolar damage, thickening of the vascular walls, and formation of microthrombi that clog capillaries. Hypercoagulable conditions accelerate the worsening of V/Q mismatch and lung tissue damage [16].

Risk factors for silent hypoxemia are old age and having diabetes [9] and these factors are known to blunt the body's regulatory response to hypoxia [17], as in our case: a 60-year-old-male with comorbidity of diabetes mellitus. Therefore, early detection of silent hypoxemia such as by using prehospital pulse oximetry [5], or radiology imaging [18,19] might provide some red flag signs of impending danger of eminent cardiac arrest or sudden respiratory failure.

## 4 Conclusions

Silent hypoxemia might be considered as an early clinical sign of deterioration of patients with COVID-19, thus, the physician may be able to intervene early and decrease its morbidity and mortality.

## Consent of patient

Written informed consent was obtained from the patient for publication of this case report and accompanying images. A copy of the written consent is available for review by the Editor-in-Chief of this journal on request.

## Provenance and peer review

Not commissioned, externally peer reviewed.

## List of Abbreviations

SARS-CoV-2: Severe Acute Respiratory Syndrome Coronavirus 2
COVID-19: Coronavirus Disease 2019
RR: Respiratory rate
ACE2: Angiotensin-converting-enzyme 2
RAAS: renin-angiotensin-aldosterone system
